# Polyphenylsulfone (PPSU)-Based Copolymeric Membranes: Effects of Chemical Structure and Content on Gas Permeation and Separation

**DOI:** 10.3390/polym13162745

**Published:** 2021-08-16

**Authors:** Fan Feng, Can-Zeng Liang, Ji Wu, Martin Weber, Christian Maletzko, Sui Zhang, Tai-Shung Chung

**Affiliations:** 1 Department of Chemical & Biomolecular Engineering, National University of Singapore, Singapore 117585, Singapore; e0554256@u.nus.edu (F.F.); chelian@nus.edu.sg (C.-Z.L.); chezhangsui@nus.edu.sg (S.Z.); 2 Integrative Sciences and Engineering Programme, NUS Graduate School, National University of Singapore, Singapore 119077, Singapore; e0145783@u.nus.edu; 3 Advanced Materials & Systems Research, BASF SE, 67056 Ludwigshafen, Germany; martin.weber@basf.com; 4 Performance Materials, BASF SE, 67056 Ludwigshafen, Germany; christian.maletzko@basf.com; 5 Graduate Institute of Applied Science and Technology, National Taiwan University of Science and Technology, Taipei 106335, Taiwan

**Keywords:** polyphenylsulfone (PPSU), tetramethylbiphenol polysulfone (TMPPSf), fractional free volume (FFV), d-spacing, gas separation

## Abstract

Although various polymer membrane materials have been applied to gas separation, there is a trade-off relationship between permeability and selectivity, limiting their wider applications. In this paper, the relationship between the gas permeation behavior of polyphenylsulfone(PPSU)-based materials and their chemical structure for gas separation has been systematically investigated. A PPSU homopolymer and three kinds of 3,3′,5,5′-tetramethyl-4,4′-biphenol (TMBP)-based polyphenylsulfone (TMPPSf) copolymers were synthesized by controlling the TMBP content. As the TMPPSf content increases, the inter-molecular chain distance (or d-spacing value) increases. Data from positron annihilation life-time spectroscopy (PALS) indicate the copolymer with a higher TMPPSf content has a larger fractional free volume (FFV). The logarithm of their O_2_, N_2_, CO_2_, and CH_4_ permeability was found to increase linearly with an increase in TMPPSf content but decrease linearly with increasing 1/FFV. The enhanced permeability results from the increases in both sorption coefficient and gas diffusivity of copolymers. Interestingly, the gas permeability increases while the selectivity stays stable due to the presence of methyl groups in TMPPSf, which not only increases the free volume but also rigidifies the polymer chains. This study may provide a new strategy to break the trade-off law and increase the permeability of polymer materials largely.

## 1. Introduction

The membrane-based separation technology is superior to other conventional processes for gas separation in terms of energy consumption, footprint, environmental impact, maintenance cost, and easy operation [1,2,3,4,5,6]. Various polymeric materials have been developed and applied to gas separation in the recent years [6,7,8,9,10,11,12,13,14,15,16]. However, challenges still exist, particularly in developing advanced polymeric membrane materials, because of the existing trade-off relationship between gas permeability and selectivity [17,18,19,20,21]. To overcome this challenge, several strategies have been proposed and studied. For example, synthesis of new polymeric materials [6,7,10,11,12,13,16,22,23,24], cross-linking [7,24,25,26], development of mixed matrix membranes [3,11,27,28,29,30,31,32], and polymer blends [9,33,34].

Among these methods, synthesizing a totally new polymer is relatively difficult. However, two breakthroughs have occurred in the last two decades; one was the polymers of intrinsic microporosity (PIMs) [10,35,36] while the other was the thermally rearranged (TR) polymers [37]. The former took the advantages of intra-chain and inter-chain rigidities to inhabit the rotation of the backbone, resulting in higher free volumes and excellent transport properties. The latter induced in situ TR of ortho-hydroxy polyamides or polyimides at elevated temperatures to produce polybenzoxazole (PBO) with higher free volumes and thermal properties. Both materials have shown promising gas separation performances because of the high permeability and specific surface area [9,38,39]. However, to our best knowledge, their gas separation membranes have not been fully commercialized yet. Although other techniques including cross-linking, nanocomposites and polymer blends have made some progresses for enhancing the separation performance of polymers, most of their membrane materials are still in the laboratory stage due to insufficiently improved performance, difficulties and costly thin-film fabrication or compromised processability and mechanical robustness. They are hence still far from commercialization.

In contrast, polyphenylsulfone (PPSU) is a commercially available amorphous high-performance thermoplastic, it has better impact resistance and chemical stability than polysulfone (PSU) and polyimide. Thus, PPSU has been extensively studied over the past several years for gas separation [40,41], organic solvent nanofiltration [42,43,44], fuel cell [45,46], and water reuse [47,48,49,50]. PPSU is part of the polysulfone family and polysulfone has been used for the early development of gas separation membranes by Air Products with the aid of silicone rubber coating [51], which is now explored in this work for its feasibility of structural modification to enhance gas separation. The relationship between chemical structure and gas performance of polysulfone have been well studied [52,53,54,55]. For example, McHattie et al. found that the polysulfone with the tetramethyl ring substitution had a higher permeability while maintaining a comparable selectivity [52]. Aitken et al. investigated the effects of symmetry and unsymmetric placements of methyl groups on polysulfone, they concluded that a unsymmetric structure in polysulfone led to a lower permeability and a higher selectivity [53]. Dai et al. synthesized tetramethyl- polysulfones with trimethylsilyl groups and reported that a higher degree of substitution led to higher CO_2_ and O_2_ permeability and an excellent CO_2_/N_2_ selectivity, which was near the Robeson upper-bound [55]. However, there are limited reports about the effects of chemical structure on gas separation and sorption properties of PPSU-based copolymers.

**Figure 1:**
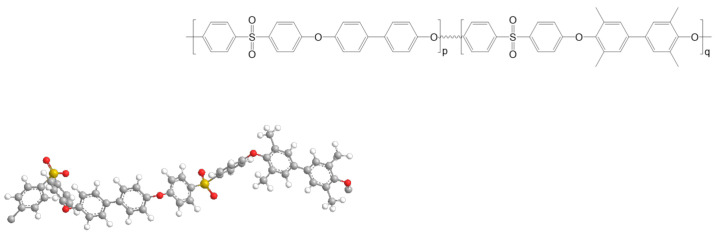
Schematic of Polyphenylsulfone/Tetramethylbiphenol polysulfone (PPSU/TMPPSf) copolymers. p represents the mole fraction of PPSU, and q represents the mole fraction of TMPPSf in copolymers (q = 0, 0.46, 0.64, or 0.74 in this work).

3,3′,5,5′-Tetramethyl-4,4′-biphenol (TMBP) was chosen in this study to modify PPSU because it has been widely used to modify polysulfone [54,55] and polyetherketones with high strength and dimensional stability [56] and gas separation performance [57]. Figure 1 shows the basic structure of our newly developed PPSU-based copolymers. The first segment is the PPSU moiety, and the second segment is the polysylfone (PSF)-based tetramethylbiphenol moiety consisting of four methyl groups attached on the benzene rings.

In this paper, we aim to investigate the relationship between the gas permeation behavior and the effects of methyl groups in PPSU-based copolymers. Four copolymers were synthesized by BASF and their dense film membranes were cast. Various techniques such as Fourier transform infrared spectrometer (FTIR), nuclear magnetic resonance (NMR), wide-angle X-ray diffraction (XRD), and positron annihilation life-time spectroscopy (PALS) were used to examine the evolution of material chemistry and microstructure with an increase in TMPPSf content. Then, the gas permeability and sorption coefficients of H_2_, O_2_, N_2_, CH_4_, and CO_2_ were measured in order to determine the effects of molecular structure on gas transport and sorption properties of dense films. This work may reveal new insights of methyl group contributions to gas transport properties of PPSU and inspire new strategies to develop novel polymeric materials for gas separations.

## 2. Experimental

### 2.1. Materials

Polyphenylsulfone (PPSU) and tetramethylbiphenol polysulfone copolymers (referred to as TMPPSf as suggested by Prof. McGrath and his co-workers [58]) were synthesized by BASF SE, Germany. All the polymers were dried in a vacuum oven at 120 °C overnight to remove the moisture. 

Figure 1 and Table 1 show their chemical structures and physical properties, respectively. The solvent dimethylformamide (DMF, HPLC grade) was bought from Sigma-Aldrich (Singapore, Singapore). All gases such as hydrogen (H_2_, ≥99.9995%), oxygen (O_2_, ≥99.9995%), nitrogen (N_2_, ≥99.9995%), methane (CH_4_, ≥99.9%), and carbon dioxide (CO_2_, ≥99.95%) were supplied by Oxygen Air Liquide Pte. Ltd. (SOXAL), (Singapore, Singapore).

### 2.2. Preparation of Dense Film Membranes

To prepare a dense film membrane, a 3 wt% polymer solution was prepared by dissolving the polymer in DMF. Then, the polymer solution was filtered using a 1 µm polytetrafluoroethylene (PTFE) filter to remove the undissolved materials. The solution was cast in a glass petri dish and dried at 120 °C in an oven for two days. Then the petri dish was further dried at 200 °C in a vacuum oven for 24 h. All dense film membranes were prepared by the casting method as described above. Each membrane thickness was measured by a Digimatic indicator (IDC-112b-5) and the average thickness of all membranes was around 50 ± 5 µm. 

### 2.3. Characterizations

A Shimadzu 50 type thermogravimetric analyser (TGA), (Shimadzu, Kyoto, Japan) was employed to analyze the thermal stability of the dense membranes. All membrane samples were heated at a speed of 10 °C/min under N_2_ atmosphere. An AccuPyc II 1340 Pycnometer (Micromeritics, Norcross, GA, USA) was utilized to measure their density. Both Fourier transform infrared spectroscopy (FTIR), (Bruker, Billerica, MA, USA) and liquid-state 400 MHz ^1^H nuclear magnetic resonance (NMR) spectroscopy (Bruker Avance III HD 400 MHz NMR Spectrometer, Billerica, MA, USA) were used to investigate the chemical structure of the polymers.

An X-ray diffractometer (XRD, Bruker D8 Advance, Billerica, MA, USA) was employed under a wide-angle X-ray diffraction (WAXD) mode to determine the inter-chain d-spacing of the polymer membranes. The XRD radiation source was Cu Kα with a wavelength of 1.54 Å and the Bragg’s rule was used to calculate the d-spacing: nλ=2dsinθ, in which n is an integer number (1, 2, 3), *λ* represents the X-ray wavelength, *d* stands for the dimension spacing and *θ* is the diffraction angle.

The fractional free volume (FFV) and pore size of the membranes were evaluated by positron annihilation lifetime spectroscopy (PALS). A variable mono-energy positron beam with a counting rate of 200–500 counts per second was used. The membranes were cut into a shape of 1 × 1 cm and the total thickness of the membrane sample was 1 mm by stacking the original dense film membranes together. The membranes were divided into two parts, 0.5 mm for each part. The ^22^Na positron source was trapped in the middle of the membranes like a sandwich: membrane–source–membrane, as described elsewhere [59]. The ortho-positronium(o-Ps) pick-off annihilation is the triplet bound state between electron and positron, which can reveal the quantitative information of the free volume and pore size.

There exists a correlation between the annihilation lifetime of o-Ps (*τ_3_* in nano-second) and the mean free volume radius *R*(Å) as stated in the semi-empirical spherical-cavity model [60,61], Equation (1)

(1) $$\tau_{3}=\frac{1}{2}{\left[1-\frac{R}{R+\Delta R}+\frac{1}{2\pi }\sin\left(\frac{2\pi r}{R+\Delta R}\right)\right]}^{-1}$$

where ∆*R* represents an empirical parameter (1.66 Å). The PALS data were analyzed by the PATFIT program, and a Gaussian distribution was applied to fit the life-time components. The fraction free volume (*FFV*) was obtained by means of the Williams–Landel–Ferry (WFL), Equation (2)

(2) $$FFV=0.0018I_{3}\upsilon_{f}\left(\tau_{3}\right)=0.0018I_{3}\left(\frac{4}{3}\pi R^{3}\right)$$

where I_3_ is the ortho-positronium intensity for *τ_3_* and *υ_f_* is the mean free volume (Å^3^) of a cavity obtained from the equivalent spherical radius R of the free volume. In addition, FFV can be estimated from the Bondi’s law based on the Van der Waals calculation as follows. Equation (3)

(3) $$FFV=\frac{V-V_{0}}{V_{0}}$$

where *V* is the specific volume obtained from the density. *V_0_* is the occupied polymer volume at 0 K. Since V_0_ and the Van der Waals volume of polymers (*V_w_*) follow the relationship of *V_o_ =* 1.3 *× V_w_*, one can obtain *V_0_* once *V_w_* is calculated from the group contribution method developed by Bondi [62]. 

### 2.4. Gas Permeation Measurements

The pure gas permeability of membranes was measured by a variable-pressure constant-volume gas permeation cell [22]. Before the tests, the membranes were vacuumed overnight in the cell. The tests were performed at a trans-membrane pressure of 2 atm at 35 °C following the order of H_2_, O_2_, N_2_, CH_4_, and CO_2_. Three samples were tested for each gas and the average was reported with a standard deviation of <10%. The gas permeability can be calculated according to the Equation (4).

(4) $$P=\frac{237\times 10^{10}}{760}\;\frac{Vl}{AT\left(P_{2}\times \frac{76}{14.7}\right)}\left(\frac{dp}{dt}\right)$$

where *P* is the membrane permeability of a gas in Barrer (1 Barrer = 1 × 10^−10^ cm^3^ (STP) cm^−2^ s^−1^ cmHg^−1^), *V* is the downstream volume (cm^3^), *l* is the membrane thickness (cm), *A* is the effective membrane area (cm^2^), *T* is the absolute temperature (K), and *p_2_* is the upstream pressure (psi). The ideal gas selectivity was defined as the ratio of permeability of two gases Equation (5).

(5) $$\alpha_{A/B}=\frac{P_{A}}{P_{B}}$$

where *P_A_* and *P_B_* are the permeability of gas A and gas B, respectively.

### 2.5. Gas Sorption Measurements

A dual-volume pressure decay method was applied to measure the sorption of pure gases by using an XEMIS microbalance apparatus [63]. Each membrane was cut into pieces of 1 × 0.5 cm and a total weight of around 120 mg was deployed for gas sorption tests. The sorption isotherms of N_2_, O_2_, and CO_2_ were performed at 35 °C with a pressure range of 0 to 10 atm. The diffusion coefficient (*D*) can be obtained from Equation (6).

(6) $$D=\frac{P}{S}$$

## 3. Results and Discussion

Among the four polymers, PPSU-0 is the homopolymer of PPSU and the other three samples, PPSU-4, PPSU-6, PPSU-7, are the copolymers of PPSU and TMPPSf, which have the additional four methyl groups. Table 1 shows the evolution of glass transition temperature (Tg (°C)), viscosity number (mL/g) and density as a function of TMPPSf content. Generally, the PPSU/TMPPSf copolymers have higher Tgs than the PPSU-0 polymer because the former has four methyl substitutions on its phenyl rings. The methyl substitutions not only reduce the chain packing but also inhabit the rotation of phenyl rings, thus increasing the chain rigidity of PPSU/TMPPSf copolymers. As a result, the density decreases with an increase with TMPPSf content, indicating that the copolymer with a higher TMPPSf content has a higher free fraction volume. 

**Figure 2:**
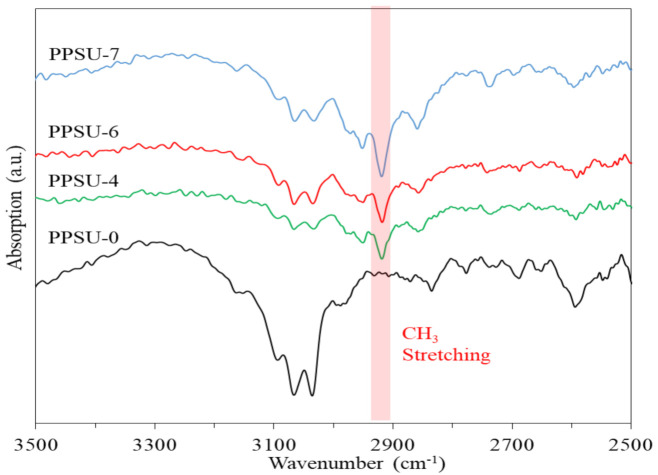
FTIR spectra of polymers.

FTIR spectra in Figure 2 display the difference between the homopolymer (PPSU-0) and the other three copolymers. The characteristic peaks at around 2960 cm^−1^ represent the stretching of methyl groups (-CH3). Such peaks only appear in the spectra of the copolymers (i.e., PPSU-4, -6, and -7) because their molecules consist of TMPPSf that contains four methyl groups. On the contrary, the homopolymer (PPSU-0) has no such peak at 2960 cm^−1^. Therefore, the molecular structures of these polymers shown in Figure 1 are confirmed. In addition, there are significant reference peaks for PPSU shown in this figure. The absorption peaks at 1155 and 1300 cm^−1^ can be ascribed to the symmetrical and asymmetrical stretching vibrations of the SO_2_ group, respectively. The peak at 1230 cm^−1^ arises from C-O stretching vibration of the ether group, and the peaks at 1486 and 1585 cm^−1^ are assigned to C-C stretching of the aromatic rings.

**Figure 3:**
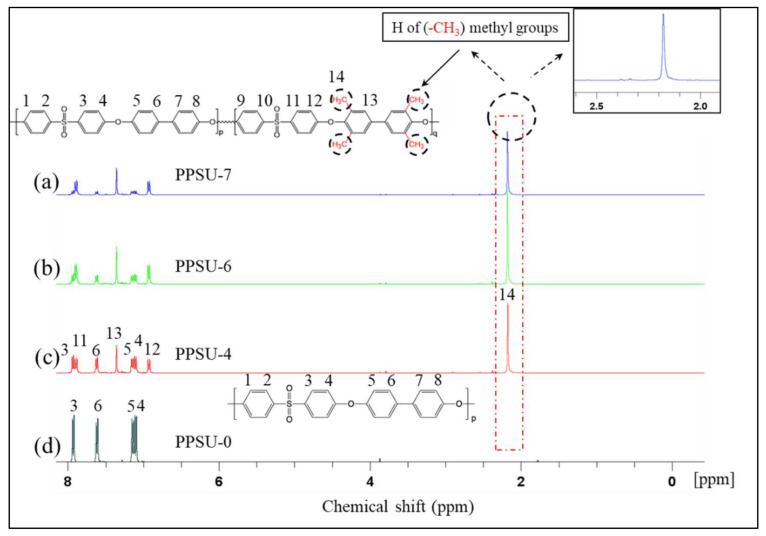
Spectra of 1H NMR of (**a**) PPSU-7, (**b**) PPSU-6, (**c**) PPSU-4, and (**d**) PPSU-0. The signal at around 2.20 ppm in spectra a, b, c reveals 1 type of proton (H) in methyl groups on the benzene rings of PPSU-4, PPSU-6, and PPSU-7.

**Figure 4:**
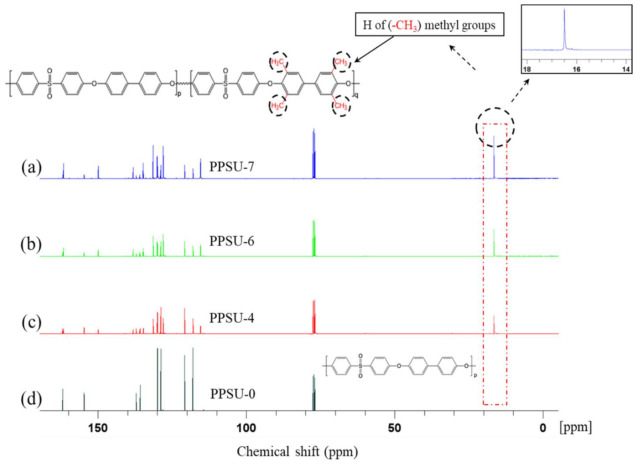
Spectra of 13C NMR of (**a**) PPSU-7, (**b**) PPSU-6, (**c**) PPSU-4, and (**d**) PPSU-0 polymers. The peaks (singlets) around 16.50 ppm in spectra a, b, c reveals 1 type of carbon (C) of symmetric methyl groups on the benzene rings of PPSU-4, PPSU-6, and PPSU-7. PPSU-0, 4, 6 and 7 are related to the TMPPSf mole fractions of 0, 0.46, 0.64 and 0.74, respectively, in copolymers, and the detailed information is listed in Table 1.

Figure 3 shows spectra of ^1^H NMR of the four polymers. It is obvious that PPSU-0 is very different from the other three polymers, especially in the signal at around 2.20 ppm, which is due to the methyl groups on the benzene rings. Figure 4 further reveals the ^13^C-NMR of the four polymers. There is a big difference around 16.50 ppm, which can be attributed to the symmetric methyl groups on the benzene rings of PPSU-7, PPSU-6, and PPSU-4. The NMR spectra further reveal the structure difference among these four polymers.

**Figure 5:**
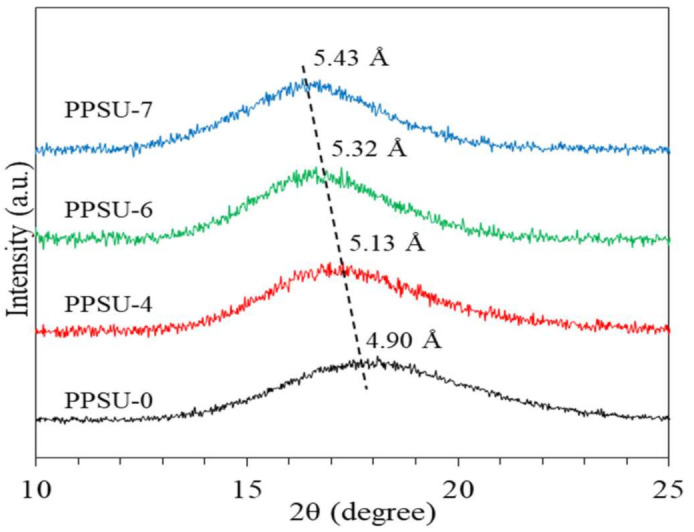
XRD spectra of polymers.

Figure 5 shows the XRD patterns and d-spacing values of these polymers. All the XRD patterns are broad because the polymers are amorphous. However, they have different d-spacings. PPSU-0 has the least d-spacing value of about 4.90 Å corresponding to a 2*θ* of 17.10 degree. The d-spacing increases with an increase in TMPPSf content. This trend is consistent with the aforementioned density data and confirms that a higher methyl content leads to a larger d-spacing in the copolymers. 

Table 2 presents the PALS results of these four polymers. By the means of the ortho positronium lifetime (τ_3_), intensity (I_3_), together with the mean free volume radius (R^3^) of each sample, the FFV can be calculated. The order of FFV follows the same trend with d-spacing: PPSU-0 < PPSU-4 < PPSU-6 < PPSU-7, which is consistent with the increasing content of TMPPSf. FFV was also calculated by the Bondi’s method and tabulated in the last column of Table 2. Both FFV results show the same trend.

**Figure 6:**
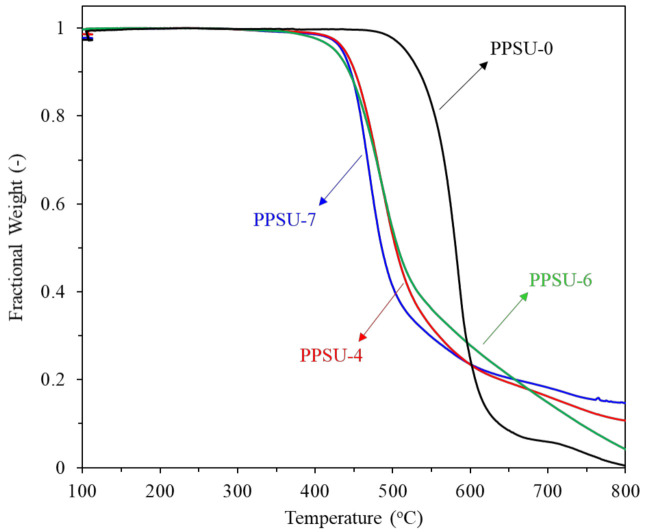
TGA spectra of polymers.

Figure 6 shows the thermal stability of the polymers by TGA. The copolymers start to decompose at around 450 °C while the homopolymer (PPSU-0) begins the decomposition at around 520 °C. Comparing to the homopolymer, the copolymers with additional methyl groups are thermally less stable because the methyl-substituted polymers are loosely packed than the homopolymer. It is known that such a loose-packed structure helps the thermal decomposition of polymers. Also, the lower thermal stability of the aliphatic units contributes to the lower thermal stability of the copolymers [64].

Table 3 summarizes the pure gas permeability and ideal selectivity of four copolymers. The gas permeability increases with an increase in TMPPSf content, while the gas selectivity remains relatively stable or slightly decreases for gas pairs such as H_2_/N_2_, O_2_/N_2_, CO_2_/CH_4_, and CO_2_/N_2_. This phenomenon results from the inhabited motion around the ether and the inhibited packing given by four methyl groups owing to the restriction of the phenyl rings for facial nesting conformation [57].

**Figure 7:**
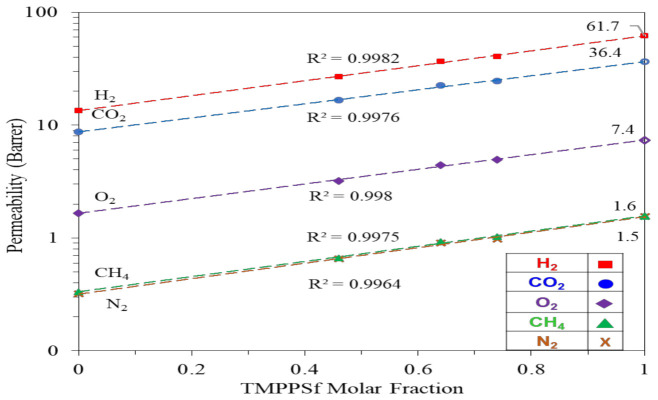
Relationship between permeability coefficient and TMPPSf content.

Figure 7 describes the relationship between permeability coefficient and TMPPSf content. There exists a linear correlation between the logarithm of permeability and the TMPPSf content. This trend fits well with the results predicted from Equation (7) [65]. In addition, one can predict the gas permeability of TMPPSf when its molar fraction is equal to 1. As shown in Table 4, the predicted data are close to the experimental results reported by others [54].

(7) $$lnP=\theta_{1}lnP_{1}+\theta_{2}lnP_{2}$$

where *P* is the permeability coefficient of the polymers, *θ*_1_ and *P*_1_ are the volume fraction and permeability of the PPSU homopolymer, respectively; *θ*_2_ and *P*_2_ are the volume fraction and permeability of the TMPPSf homopolymer, respectively. Equation (7).

**Figure 8:**
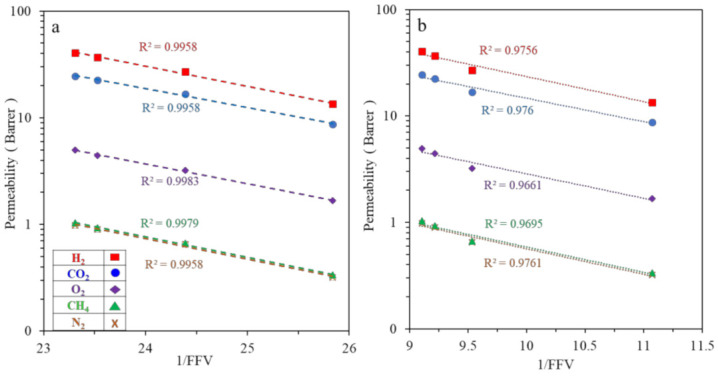
Relationship between permeability coefficient and 1/FFV. (**a**) FFV from PALS, (**b**) FFV calculation from Bondi’s way.

Figure 8 shows the relationship between the logarithm of permeability and 1/FFV for various gases using both FFV obtained from PALS and Bondi methods. The permeability declines linearly as 1/FFV increases. This trend is consistent with those TMBP modified polysulfone [54,55] and polyetherketones [57]. Clearly, the incorporation of TMPPSf into PPSU has similar effects (i.e., increased free volume and chain rigidity) as the TMBP incorporated polysulfone and polyetherketones. 

**Figure 9:**
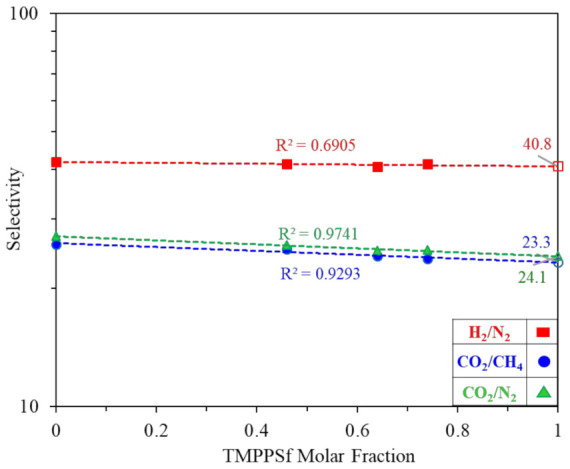
Relationship between selectivity coefficient and TMPPSf content.

The logarithm of selectivity as a function of TMPPSf content is shown in Figure 9. The dashed lines are calculated from Equation (8) using the experimental data tabulated in Table 4.

(8) $$ln\frac{P_{A}}{P_{B}}=\theta_{1}ln{\left(\frac{P_{A}}{P_{B}}\right)}_{1}+\theta_{2}ln{\left(\frac{P_{A}}{P_{B}}\right)}_{2}$$

where, *θ*_1_ and *θ*_2_ are the volume fractions of two homopolymers, respectively, while *P_A_* and *P_B_* are the permeability coefficients of two homopolymers, respectively. Equation (8).

As shown in Figure 9, the logarithm of selectivity of each gas pair from our experiments follows a linear relationship with TMPPSf molar fraction, as described in Equation (8). Therefore, one can predict the gas selectivity of TMPPSf when its molar fraction is equal to 1. The predicted data are comparable with the experiment results reported by others [54] in Table 4.

**Figure 10:**
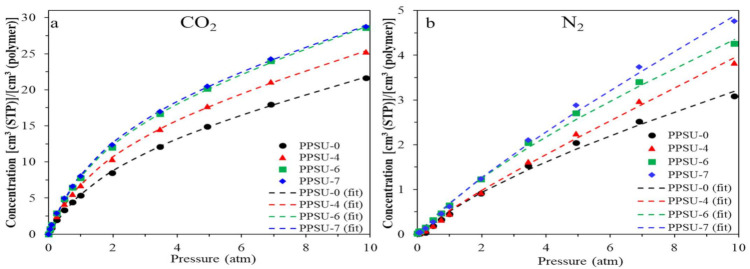
Sorption isotherms at 35 °C for PPSU-0, -4, -6, and -7: (**a**) CO_2_, (**b**) N_2_.

Figure 10 shows a comparison of sorption isotherms of CO_2_ and N_2_ at 35 °C as a function of applied pressure for PPSU/TMPPSf polymers. For each gas, at least two runs were done and the errors were within 10%. Generally, consistent with d-spacing and FFV values, the incorporation of TMPPSf into PPSU results in higher gas sorption and PPSU-7 possesses the highest sorption performance. Among all gases studied (i.e., N_2_, O_2_, CH_4_, and CO_2_), the amount of CO_2_ sorption is the highest because it is the most highly condensable one. Table 5 compares the calculated diffusion coefficients of N_2_ and CO_2_ from equation 6. Compared to N_2_, CO_2_ has a higher diffusivity because the latter has a smaller kinetic diameter than the former (i.e., 3.30 vs. 3.64 Å). All polymers display a higher gas diffusivity with an increase in TMPPSf content. Thus, the increase in gas permeability of copolymers arises from the increases in both sorption coefficient and gas diffusivity. 

**Figure 11:**
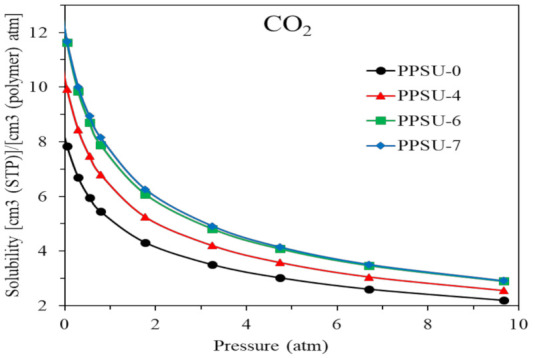
Solubility coefficient of CO_2_ as a function of pressure for PPSU-0, -4, -6, and 7.

Figure 11 plots the solubility coefficient of CO_2_ as a function of pressure and the trend follows the typical dual-mode sorption model as follows [2,16,52,53,54,55,66] Equation (9).

(9) $$C=K_{D}P+\frac{{C^{\prime }}_{H}bp}{1+bp}$$

where *C* is the gas concentration (cm^3^ (STP)/ cm^3^ membrane), *K_D_* represents the Henry law coefficient (cm^3^(STP)/ cm^3^ membrane bar), *C’_H_* and b are the Langmuir capacity parameter (cm^3^ (STP) /cm^3^ membrane), and Langmuir hole affinity (bar^−1^), respectively. 

Table 6 summarizes the calculated dual-mode parameters for CO_2_. All polymers have almost the same b values (i.e., the ratios of adsorption to desorption) of about 0.5–0.6 because they have similar chain structures, while PPSU-7 has the largest *C’_H_* among the four polymers. Also, the dual-model fitting equation in Figure 10 matches the sorption experiment data well, which is consistent with glassy polymer sorption behavior [66].

## 4. Conclusions

A series of TMBP based PPSU polymers with different TMBP content have been synthesized and their physical characteristics and gas separation performance have been investigated. It was found that as the TMPPSf content increases, the resultant copolymers have a larger d-spacing value, FFV, and sorption capacity. Thus, the logarithm of their O_2_, N_2_, CO_2_, and CH_4_ permeability increases proportionally with an increase in TMPPSf content. The enhanced gas permeability arises from the increases in both sorption coefficient and gas diffusivity of copolymers. To our delight, the selectivity stays relatively stable due to the presence of methyl groups in TMPPSf, which not only increases the free volume but also stiffens the polymer chains and inhibits their packing. This study may provide a new strategy to break the trade-off relationship between permeability and selectivity and increase the permeability of polymer materials.

## Figures and Tables

**Table 1:** Physical properties of copolymers.

Membrane	Mol% (TMPPSf)	Viscosity Number (mL/g)	T_g_ (°C)	Density (g/cm^3^)
PPSU-0	0	71.8	222	1.38
PPSU-4	0.46	62.0	246	1.32
PPSU-6	0.64	58.8	255	1.30
PPSU-7	0.74	62.2	258	1.29

**Table 2:** PALS data of membranes.

Membrane	Mol% (TMPPSf)	τ_3_ (ns)	I_3_ (%)	R_3_ (Å)	FFV (%)	FFV (%) (Bondi)
PPSU-0	0	2.00	22.00	2.86	3.87	9.03
PPSU-4	0.46	2.16	20.27	3.00	4.10	10.49
PPSU-6	0.64	2.24	19.54	3.0667	4.25	10.85
PPSU-7	0.74	2.25	19.65	3.0719	4.29	10.98

**Table 3:** Pure gas permeation properties of copolymers (tested at 35 °C and a transmembrane pressure of 2 atm, less than 10% deviation).

Membrane	Permeability (Barrer) ^a^					Ideal Selectivity				
	H_2_	O_2_	N_2_	CH_4_	CO_2_	H_2_/N_2_	O_2_/N_2_	CO_2_/CH_4_	CO_2_/N_2_	H_2_/CH_4_
PPSU-0	13.41	1.67	0.32	0.34	8.69	41.9	5.2	25.9	27.1	39.4
PPSU-4	26.96	3.21	0.65	0.67	16.74	41.4	4.9	25.1	25.7	40.2
PPSU-6	36.70	4.43	0.90	0.93	22.43	40.8	4.9	24.1	24.9	39.5
PPSU-7	40.6	4.96	0.98	1.03	24.5	41.4	5.1	23.8	25.0	39.4

^a^ 1 Barrer = 1 × 10^−10^ cm^3^ (STP) cm/cm^2^ × s × cmHg.

**Table 4:** Predicted and experiment results (measured at 35 °C under 2 atm) of gas permeability and selectivity when the TMPPSf molar fraction = 1.

Membrane TMPPSf = 1	Permeability (Barrer) ^a^					Ideal Selectivity			
	H_2_	O_2_	N_2_	CH_4_	CO_2_	H_2_/N_2_	O_2_/N_2_	CO_2_/CH_4_	CO_2_/N_2_
Predicted ^a^	61.7	7.4	1.6	1.6	36.4	40.8	4.9	23.3	24.1
Experiment ^b^	/	5.8	/	/	31.8	/	4.8	25	/

^a^ Predicted in this work. ^b^ Cited from the previous publication [54].

**Table 5:** Diffusion coefficients and solubility coefficients of PPSU/TMPPSf polymers samples.

Membrane	Diffusivity ^a^		Solubility [cm^3^ (STP)]/[cm^3^ (polymer) atm]	
	N_2_	CO_2_	N_2_	CO_2_
PPSU-0	52.78	153.35	0.45	4.25
PPSU-4	105.36	242.52	0.46	5.18
PPSU-6	109.97	280.13	0.61	6.01
PPSU-7	119.34	297.09	0.62	6.19

^a^ Diffusivity = 1 × 10^−10^ cm^2^ s^−1^. The data were measured at 2 atm and 35 °C.

**Table 6:** Dual-mode sorption model for CO_2_.

Membrane	Henry’s Law Coefficient	Maximum Sorption Capacity	Langmuir Hole Affinity
	*K_D_* [(cm^3^) (STP)]/ [cm^3^ (polymer) atm]	*C′_H_* [(cm^3^) (STP)]/ [cm^3^(polymer)]	*b* (1/atm)
PPSU-0	1.086	13.09	0.502
PPSU-4	1.228	15.41	0.567
PPSU-6	1.360	17.71	0.588
PPSU-7	1.294	18.72	0.576

## Data Availability

Not applicable.
